# Translation, cultural adaptation, validation and reliability of Persian version of Edmonton frailty score questionnaire among Iranian heart failure individuals

**DOI:** 10.22088/cjim.14.1.53

**Published:** 2023

**Authors:** Hojatollah Alaei, Mehrbod Vakhshoori, Maryam Heidarpour, Farbod Khanizadeh, Niloofar Bondariyan, Sayed Ali Emami, Awat Feizi, Davood Shafie

**Affiliations:** 1 Heart Failure Research Center, Isfahan Cardiovascular Research Institute, Isfahan University of Medical Sciences, Isfahan, Iran; 2 Isfahan Endocrine and Metabolism Research Center, Isfahan University of Medical Sciences, Isfahan, Iran; 3 Insurance Research Center, Tehran, Iran; 4 Department of Clinical Pharmacy, School of Pharmacy, Shiraz University of Medical Sciences, Shiraz, Iran; 5 Department of Epidemiology and Biostatistics, Isfahan University of Medical Sciences, Isfahan, Iran; ¥ “Hojatollah Alaei” and “Mehrbod Vakhshoori” contributed equally in this manuscript and are considered to be co-first authors.

**Keywords:** Heart failure, Frailty, Validation study, Reproducibility of results, Iran

## Abstract

**Background:**

Frailty is a common problem in elderly individuals. However, this issue is not well investigated among heart failure (HF) patients with appropriate scales. We aimed to translate and evaluate Edmonton frailty scale (EFS) validity and reliability in Iranian HF adults.

**Methods:**

We implemented this methodological study on stable HF patients referred to an outpatient heart clinic in Isfahan, Iran. The translation was done using the forward-backward method. Ten individuals were asked to comment about all items in terms of understandability and simplicity. Fifteen experts were invited, and their ratings on each item were collected to measure the content validity index (CVI) and content validity ratio (CVR). Cronbach’s alpha was used for the assessment of internal consistency. After completing the scale for the second time with a two-week interval, test-retest reliability with intraclass correlation coefficient (ICC) measurement was done.

**Results:**

The translation process was performed uneventfully. All items were reported to be simple and meaningful. CVI of items ranged from the minimum of 0.80 to a maximum of 1.00 plus an acceptable CVR of at least 0.60. Fifty HF patients (age: 67.2±14.1 years, males: 56%) completed the questionnaire twice without missing data. Cronbach’s alpha was first to be 0.550. After omitting three items about social support, drug usage, and nutrition, the value was raised to 0.711. Test-retest reliability showed a good index of consistency (ICC: 0.693, 95% confidence interval: 0.527-0.810).

**Conclusion:**

Modified Persian EFS is a simple and meaningful tool with high validity and acceptable reliability for assessing frailty in HF individuals irrespective of age.

One of the most common cardiovascular diseases (CVDs) in the elderly population is heart failure (HF). This complex disease with different etiologies and clinical manifestations has been one of the leading causes of mortality and morbidity in developed nations (1). Although HF prevalence among the adult population is estimated to be 1-2%, this prevalence constantly increases with aging. HF prevalence among men aged 60-79 and more than 80 years is being reported to be 6.6% and 10.6%, respectively. This prevalence is reported to be 4.8% and 13.5% among females with the same age categories as previously mentioned males (1-3). Despite numerous improvements in HF management, 5-year mortality and re-hospitalization rates have been announced to be 50% and 20-25%, respectively (1, 4). Despite numerous improvements in HF management, 5-year mortality and re-hospitalization rates have been announced to be 50% and 20-25%, respectively (1, 4). Moreover, the tremendous economic burden caused by this entity should be considered. The annual expenditure of HF management has been ranged from $908 to $40971 for each patient (5). 

Frailty is a general term indicating a reduced physiologic reserve and increased susceptibility to various endogenous and exogenous stress stimuli, consequently leading to heightened death and re-hospitalization rates (6-8). Two basic approaches, including physical frailty phenotype and cumulative deficit model, have been declared to assess frailty. The former was suggested by Freid and colleagues characterized as a physical syndrome with five criteria including weak grip strength, unintentional weight loss, exhaustion, slow walking speed, and low physical activity (9). *Rockwood et al.* defined the latter approach as a complex syndrome that consisted of physical and non-physical health issues (10).

This syndrome is more prevalent among HF patients with an estimated rate of 45% (6). Furthermore, the dimension of HF Association (HFA), affiliated with the European society of cardiology (ESC), declared a scientific definition of frailty among HF patients as a “*multi-dimensional dynamic state independent of age that makes the individuals with HF more vulnerable to the effects of stressors” *(6). There are several available screening and assessment tools for frailty evaluation, including Derby frailty index (DFI), acute frailty network (AFN), clinical frailty scale (CFS), Fried frailty phenotype, deficit index (DI), and Edmonton frailty scale (EFS) (11). 

The latter is a simplified assessment tool with multiple categories: cognition, general health status, functional dependence, social support, medication use, nutrition, mood, continence, and functional performance (12). Although EFS has been validated previously, usages of this questionnaire among different nations with different languages might be limited. This study aims to assess Persian translating as well as the validity and reliability of EFS among Iranian HF individuals.

## Methods

**Edmonton Frailty Scale: **EFS is an 11-item questionnaire with nine frailty domains, including cognition, general health status, functional dependence, social support, medication use, nutrition, mood, continence, and functional performance (12). Six items in the questionnaire have Likert-type answer choices, and the other five questions are two-scale answer choices (yes/no). Two domains, including the cognition and functional performance section, are performance-based items. The minimum and maximum overall scores in this questionnaire range from 0 to 17. EFS scores of 0-5, 6-7, 8-9, 10-11, and 12-17 are defined as no frailty, vulnerable, mild, moderate, and severe frailty, respectively. 

**Translation:** The translation process is performed in 5 stages, as suggested by *Beaton et al.’s* study (13). At first, the original questionnaire was translated from English to Persian by two independent translators who were native Farsi speakers and fluent in English. One of the translators was familiar with medical terms, and the other was not. They were told to use simple, understandable words rather than scientific terms. Next, a consensus was made between two translators on the first translated version. In the third phase, the Persian questionnaire was translated back to English. A pre-final version of the questionnaire was created in a meeting with translators and a methodologist in the next stage. Finally, this pre-defined questionnaire was distributed among 10 patients with HF. Each participant fulfilled the questionnaire in the presence of the principal investigator and declared his/her understanding from each item in the questionnaire. Moreover, any doubt on questions about each questionnaire item (either provided questions or answers) was collected. All authors reviewed all comments and suggestions for possible modification of questionnaire items.

**Validity:** In order to evaluate the face validity of the questionnaire, a group of experts, including six cardiologists, two general practitioners, one pharmacist, five nurses, and one statistician, were invited. They were asked to read each item in the questionnaire and declare their opinions about comprehensibility and understandability as well as relevance. Davis technique was used to calculate content validity index (CVI) as the following: 1: not suitable, 2: suitable in terms of readapting prepositions, 3: suitable but some adaptations are required, and 4: very suitable. Division of the number of experts rated 3 or 4 for each item by the total number of experts defined CVI. The overall score of at least 0.80 was considered acceptable. For each item in the questionnaire, a CVI score of more than 0.79, 0.70 to 0.79, and less than 0.70 were rated as appropriate, requiring re-evaluation and candidate for removal, respectively (14, 15). Moreover, all experts were asked to use a three-item scale including terms like “essential”, “important, but not essential,” and “not essential” to rate each questionnaire item for calculation of content validity ratio (CVR). The minimum acceptable CVR value was defined to be 0.60 (16). 

**Reliability: **Cronbach’s alpha coefficient was used for the determination of the internal consistency of the questionnaire. Therefore, the translated questionnaire was randomly distributed among 50 patients who suffered from HF. Cronbach’s alpha of more than 0.9 was defined as excellent. The following values were defined as other status of internal consistency: good: > 0.8, acceptable: > 0.7, questionable: > 0.6, poor: > 0.5 and unacceptable: < 0.5. We considered a Cronbach’s alpha between 0.7 and 0.9 as proposing good reliability. In order to assess test-retest reliability, HF patients were asked to complete the questionnaire two times at a 2-week interval. Interclass correlation coefficients (ICC) of ≥ 0.75, 0.4 to 0.75, and ≤ 0.4 were considered as excellent, fair to good and poor reliability, respectively (14, 17).

**Floor and ceiling effects**: Floor and ceiling effects were recorded when at least 15% of participants got the lowest and highest scores, respectively.

**Statistical analysis: **Statistical Package for Social Sciences (SPSS) version 26 (IBM Corp., Armonk, NY, USA) was used to perform all analyses. Frequency (percentage) and mean ± standard deviation (SD) were utilized to report the distribution of total scores.

**Ethical consideration: **This study was approved by the ethics committee affiliated with Isfahan University of Medical Sciences (IUMS) (IR.MUI.MED.REC.1400.173). All participants were fully explained about the study and its objectives, and any probable questions were answered thoroughly, and each individual signed a written consent form. Moreover, they were told that all personal information, including names or any other identical documents, was kept confidential and not disclosed publicly.

## Results

**Participants’ characteristics: **We randomly selected 50 patients with documented HF referred to an outpatient heart clinic in Isfahan, Iran (Charmran heart clinic) from March-May 2021. All recruited patients were literate and we discarded any subjects who was illiterate. The mean age of our study sample was 67.2 ± 14.1 years (males: 56%). All patients thoroughly completed the questionnaire. Moreover, they were invited to come back to the clinic to complete the questionnaire for the second time. The same investigator in both sessions assessed items 1 and 11. The distribution of each item's answer choices is shown in Table 1. The mean frailty scores in the first and second completion times were 8.74±2.12 and 8.12±2.03, respectively. 4% of enrolled participants had no frailty, and 14% were vulnerable to frailty. The highest prevalence was attributed to mild frailty (44%). However, 34% and 4% suffered from moderate and severe frailty, respectively.

**Table 1:** Distribution of respondents’ answers during the first and second time of Edmonton frailty scale (EFS) completion (n= 50)

Questions	First time			Second time		
	Point 0 (%)	Point 1 (%)	Point 2 (%)	Point 0 (%)	Point 1 (%)	Point 2 (%)
**Item 1**	14 (28)	32 (64)	4 (8)	14 (28)	32 (64)	4 (8)
**Item 2**	10 (20)	24 (48)	16 (32)	5 (10)	29 (58)	16 (32)
**Item 3**	2 (4)	41 (82)	7 (14)	10 (20)	40 (80)	0
**Item 4**	2 (4)	8 (16)	40 (80)	2 (4)	8 (16)	40 (80)
**Item 5**	44 (88)	6 (12)	0	44 (88)	6 (12)	0
**Item 6**	4 (8)	46 (92)	-	4 (8)	46 (92)	-
**Item 7**	42 (84)	8 (16)	-	42 (84)	8 (16)	-
**Item 8**	37 (74)	13 (26)	-	46 (92)	4 (8)	-
**Item 9**	4 (8)	46 (92)	-	4 (8)	46 (92)	-
**Item 10**	45 (90)	5 (10)	-	44 (88)	6 (12)	-
**Item 11**	1 (2)	24 (48)	25 (50)	3 (6)	33 (66)	14 (28)
**Total score**	8.74 ± 2.12			8.12 ± 2.03		

**Figure 1:**
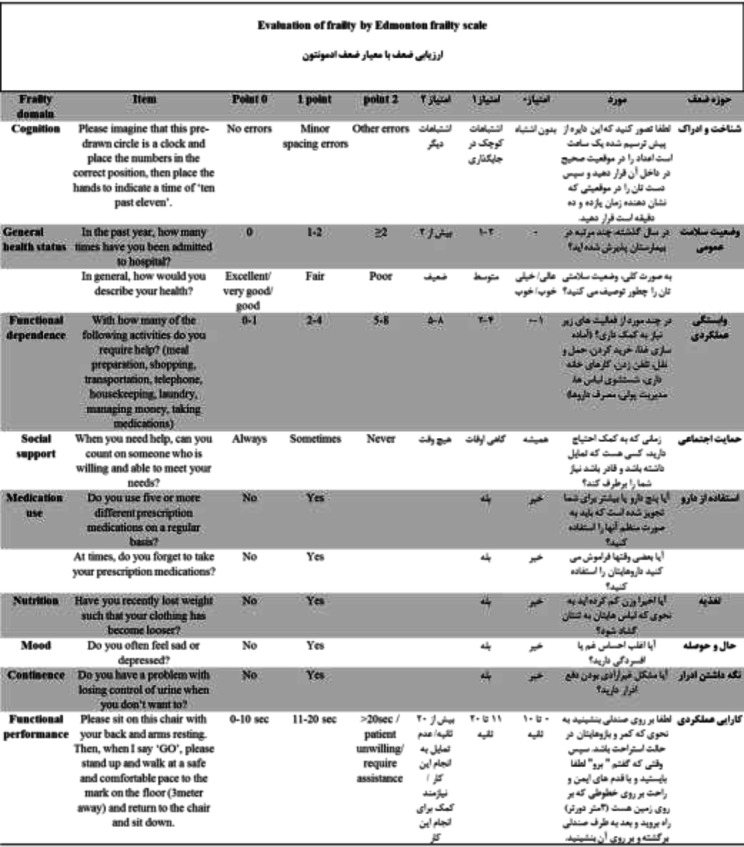
Persian and original version of Edmonton frailty scale

**Translation: **From 10 different HF patients recruited for announcing their comments on each questionnaire item, all individuals told the questions were easily understandable with no subsequent further significant changes. The final Persian version of EFS in comparison to the original scale is shown in Fig. 1.

**Scale validity: **The validity indices of each questionnaire item are presented in Table 2. CVI of items ranged from 0.80 to 1.00. Also, all items showed acceptable CVR (minimum: 0.60, maximum: 1.00).

**Scale reliability: **The Cronbach’s alpha of all included questions was 0.550. after the omission of three items (item 5 on social support, item 6 on medication usage, and item 8 on nutrition), this value rose considerably and reached an acceptable level of 0.711. After completion of questionnaire for two times, the ICC with consideration of remained items was found to be 0.693 (95% confidence interval (CI): 0.527-0.810). Table 3 presents the results of reliability indices of translated questionnaire items after the omission of undesirable questions. Corrected item-total correlation (CITC) values ranged from 0.201 to 0.687. The final version of Persian EFS is depicted in the supplementary appendix (page 59).

**Floor and ceiling effects: **None of our respondents got the highest or the lowest scores; thus, floor and ceiling effects were not observed. 

**Table 2:** Validity indices of the Persian version of Edmonton frailty scale (EFS)

Questions	Content validity index	Content validity ratio
**Item 1**	1.00	0.60
**Item 2**	0.93	0.73
**Item 3**	1.00	0.73
**Item 4**	0.93	0.60
**Item 5**	0.80	0.73
**Item 6**	0.93	0.87
**Item 7**	0.86	1.00
**Item 8**	1.00	0.73
**Item 9**	0.86	0.87
**Item 10**	0.80	1.00
**Item 11**	1.00	0.60

**Table 3:** Reliability indices of the Persian version of Edmonton frailty scale (EFS) after omission of three items (items 5, 6, and 8)

Questions	Corrected item-total correlation	Cronbach's alpha if item deleted
**Item 1**	0.477	0.665
**Item 2**	0.250	0.744
**Item 3**	0.526	0.661
**Item 4**	0.687	0.613
**Item 7**	0.201	0.717
**Item 9**	0.581	0.671
**Item 10**	0.229	0.712
**Item 11**	0.489	0.662

## Discussion

The main aim of the current research was the assessment of the validity and reliability of the Persian version of EFS. Our data suggested that after the omission of three items, the internal reliability of the scale increased to an acceptable level. Since frailty is one of the HF patients' bothersome symptoms and negatively affects individuals’ quality of life, the proper diagnosis could aid physicians in implementing better interventions. EFS was first developed by *Rolfson et al.* to assess frailty among geriatric patients. They enrolled 158 individuals and found this scale is a valid and reliable tool capable of frailty evaluation in elderly subjects (12).

In the current study, we decided to use the forward-backward translation method rather than the dual-panel way. Although both methods are practical, several differences exist.

 In dual-panel, two groups, including bilingual and lay panel, perform the translation process. The former group contains bilingual individuals translating the preliminary version of the questionnaire. The latter group consists of monolingual persons with different educational levels and socioeconomic backgrounds. The main responsibility of the lay panel is the assessment of the translated questionnaire for understandability and comprehensiveness (18, 19). This method is time-consuming and difficult to implement. Moreover, it has been recommended that the translation resulted from this method should be checked with backward translation (20, 21). Thus, the decision was made to use the forward-backward method. By using this method, no missing item was found in our study, and respondents' comments favored the simplicity and understandability of all items. Also, participants reported that the minimum required time for completing the questionnaire was less than five minutes, comparable to the original version (12). 

The first Cronbach’s alpha of our translated scale was quite low (0.550). However, the deletion of three items related to social support, medication usage, and nutrition resulted in increased internal consistency to an acceptable level. The Cronbach’s alpha of the original EFS was 0.62 (12). The Polish draft of EFS on 382 inpatient geriatric patients showed a Cronbach’s alpha of 0.709. However, its study sample consisted of those with the stable chronic disease during hospitalization (22). One hundred thirty elderly individuals administered the Turkish translated EFS in the nursing home, and the Cronbach’s alpha was found to be 0.75 with CITC values ranged 0.12 to 0.65 (23). They suggested this tool could be reliably measured frailty among Turkish elderly subjects. Enrollment of healthy elderly adults with no apparent diseases should be considered for their reported findings. The test-retest reliability correlation after questionnaire completion by 30 individuals two times after 2-3 weeks interval revealed a significant correlation (r: 0.98, P< 0.001) (23). Possible explanations for the low Cronbach’s alpha resulting from all included items might be due to cultural issues or implementing this scale on a specific group of patients. Our data showed that all included items had a high index of validity, and this scale assessed accurately a feature that was intended to be measured. Furthermore, the Turkish and Portuguese versions of this scale approved the validity of EFS in their nations (23, 24). 

To the best of our knowledge, this study is the first in the literature investigating the cultural adaptation and validity as well as reliability of EFS among patients with HF regardless of age. All recruited individuals fulfilled the questionnaire twice with no missing data. However, some limitations are still present. This study was performed in one center. Therefore, our findings should be deducted cautiously for generalization to other Iranian HF patients living in other cities and it might be considered as a regional questionnaire. Quite small sample size could be categorized as another limitation and might reduce the generalizability of our findings. Although all patients were literate, we did not evaluate their educational degree. This aforementioned factor might affect our outcomes.

In conclusion, this study indicates that modified Persian translated EFS is a reliable and valid instrument for assessing frailty among Iranian HF sufferers with no age limitation and might be a practical tool in the clinical environment. Other studies in other nations are required to establish the validity and reliability of this tool in HF. 
